# Sexual Dimorphism and Population Variation in Median Nerve Morphometry: A Multi-Institutional Cadaveric Analysis

**DOI:** 10.3390/diagnostics16111719

**Published:** 2026-06-03

**Authors:** Jessica Nguyen, Alfredo A. Torres-Rodriguez, Mario Loomis, Sofia Jimenez-Dietsch, Norman Ramirez-Lluch, Heather F. Smith, Jailenne I. Quiñones-Rodríguez

**Affiliations:** 1 College of Osteopathic Medicine, Sam Houston State University, Conroe, TX 77304, USA; 2 Department of Clinical Anatomy, College of Osteopathic Medicine, Sam Houston State University, Conroe, TX 77304, USA; 3 Department of Anatomy and Cell Biology, Universidad Central del Caribe School of Medicine, Bayamon, PR 00956, USA; 4 Department of Pediatric Orthopedic Surgery, Mayagüez Medical Center, Mayagüez, PR 00681, USA; 5 Department of Anatomy, Midwestern University, Glendale, AZ 85308, USA; 6 School of Human Evolution and Social Change, Arizona State University, Tempe, AZ 85308, USA

**Keywords:** median nerve, anatomical variation, sexual dimorphism, peripheral nerve, cadaveric dissection, morphometry, clinical anatomy

## Abstract

**Background/Objectives:** The median nerve exhibits clinically relevant anatomical variability, with critical implications for surgical exposure, regional anesthesia, peripheral nerve repair, and diagnostic imaging. Despite extensive descriptive reports, quantitative morphometric analyses incorporating biological sex and donor record population classification remain limited, particularly among Hispanic cadaveric cohorts. **Methods:** This multi-institutional cadaveric study evaluated median nerve formation and segmental morphometry in 82 anatomical donors, each contributing paired bilateral upper limbs (164 upper limbs total), classified in institutional records as Puerto Rican, Caucasian, or African American. Standardized dissections were performed from the brachial plexus to the distal forearm, and linear measurements were obtained between predefined anatomical landmarks using digital calipers. **Results:** Mixed-effects modeling was used to evaluate morphometric differences associated with biological sex, donor record population classification, and anatomical location. Male specimens demonstrated localized differences in proximal median nerve formation, including a longer medial cord contribution on the left side. Population group comparisons identified greater measured morphometric distances in the Puerto Rican cohort compared with Caucasian and African American cohorts. Normalized LEH/MEH ratios did not differ between sexes, suggesting that some absolute differences may reflect limb-size scaling rather than proportional shifts in nerve position. **Conclusions:** These findings provide standardized landmark-based morphometric data and support the value of population-sensitive anatomical reference data for surgical planning and imaging-based interpretations of median nerve morphology.

## 1. Introduction

Anatomical variations of the median nerve are frequently encountered and carry substantial clinical and surgical implications. These variations influence the diagnosis and management of peripheral neuropathies, including carpal tunnel syndrome and pronator teres syndrome, and may complicate procedures in which the median nerve is exposed or manipulated [1]. During carpal tunnel release, one of the most commonly performed hand surgeries worldwide, unrecognized transligamentous or high-division (bifid) median nerve configurations can result in incomplete decompression or iatrogenic injury to the thenar motor branch, potentially leading to motor dysfunction or sensory deficits [2]. Similarly, during thenar reconstruction or palmar flap elevation, superficial or accessory branches may be vulnerable to inadvertent transection, thereby compromising functional outcomes [3]. From an orthopedic perspective, deviations in median nerve trajectory or the presence of a persistent median artery may increase the risk of traction, compression, or entrapment during volar plating of distal radius fractures [4]. Accordingly, precise anatomical characterization of median nerve variability is essential for operative planning and complication avoidance.

Anatomically, the median nerve is formed by contributions from the lateral and medial cords of the brachial plexus (C5-T1) [5,6]. It courses through the anterior compartment of the arm, passes medial to the brachial artery within the cubital fossa, and enters the forearm between the flexor digitorum superficialis and flexor digitorum profundus muscles [7,8]. In the forearm, it gives rise to the anterior interosseous nerve, which innervates the deep flexor compartment [9], and provides palmar cutaneous sensory branches. At the wrist, the median nerve traverses the carpal tunnel deep to the flexor retinaculum and divides into terminal motor and sensory branches supplying the thenar musculature and lateral digits [10,11]. Although this classical trajectory is well established, considerable variability has been documented in both proximal formation and distal branching.

Among these variants, the thenar motor branch is the most clinically relevant. Poisel categorized the thenar motor branch patterns according to their relationship with the flexor retinaculum as extraligamentous, subligamentous, or transligamentous [11]. Additional preligamentous variants have also been described [12,13,14,15]. Lanz subsequently expanded this classification to include accessory branches, high divisions of bifid median nerve, and variable communication patterns [14,15,16,17,18]. Because these branches lie within operative corridors, accurate identification of their course is critical during decompression, reconstruction, and microsurgical procedures to prevent irreversible thenar muscle denervation and functional impairment.

**Figure 1:**
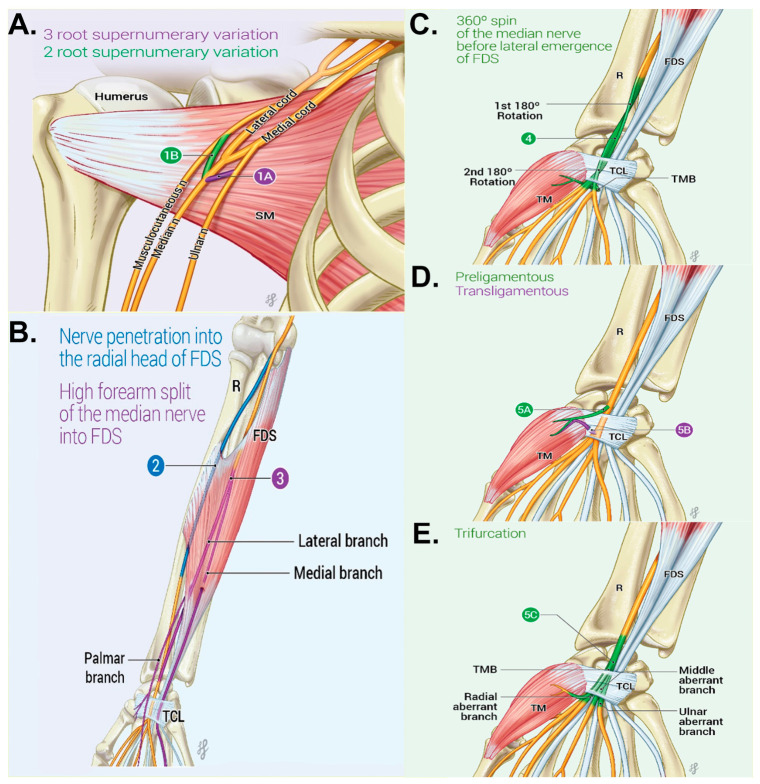
Anatomical variations of the median nerve (MN) across the upper limb. Schematic illustration summarizing categorized anatomical variations of the MN from proximal formation in the brachial plexus to distal branching at the wrist. Numbered labels (1–5) indicate major variation categories, with sublabels identifying specific patterns within each category. ((**A**) Category 1) Brachial plexus root variations showing supernumerary root contributions to the MN from the lateral and medial cords adjacent to the subscapularis muscle (SM) and proximal humerus. ((**B**) Category 2) Proximal forearm bifurcation of the MN, including penetration of the MN into the radial head of the flexor digitorum superficialis (FDS) along the radius (R), followed by early division into medial and lateral branches. ((**C**) Category 4) Rotational variation of the MN demonstrating sequential 180° rotations as the nerve courses distally toward the wrist before passing beneath the transverse carpal ligament (TCL). ((**D**) Categories 5A and 5B) Preligamentous and transligamentous thenar motor branch (TMB) variations, in which the TMB arises proximal to, or passes through, the TCL before entering the thenar muscles (TM). ((**E**) Category 5C) Distal MN trifurcation beneath the TCL, producing radial, middle, and ulnar aberrant branches supplying the TM and distal hand structures. Illustration reproduced with permission from Lucia Garces, © 2026.

Beyond these well-recognized patterns, median nerve variability has been documented throughout the upper limb. Variations may occur proximally at the level of the brachial plexus, within the forearm, or distally at the wrist and carpal tunnel (Figure 1). Proximal variations commonly involve supernumerary root contributions from the lateral or medial cords and atypical fusion patterns during formation of the median nerve (Figure 1A) [5,6,19,20]. Quantitative analyses of published datasets suggest potential demographic variability. However, interpretation is limited by inconsistent reporting of biological sex and heterogeneous study designs [5,6,19,20]. Geographic comparisons further demonstrate variability in reported frequencies, with studies from South Asian populations reporting higher frequencies, 25% to 34% in moderate-sized cadaveric cohorts [6,19,20]. Intermediate frequencies have been described in Russian populations [5], whereas larger European imaging and cadaveric studies have reported lower frequencies, typically below 10% [21,22]. In contrast, isolated case reports may describe high apparent frequencies of variation, but these typically reflect small sample sizes or multiple concurrent variations within individual specimens rather than true population prevalence [20].

Regionally, forearm variants include high division, accessory branches, and muscular penetrations such as passage through the radial head of the flexor digitorum superficialis (Figure 1B) [23,24]. At the wrist and carpal tunnel, clinically relevant variants include recurrent thenar motor branch variation, bifid median nerve, persistent median artery, and distal branching anomalies such as trifurcation patterns (Figure 1C–E) [13,16,17,18,21,22]. Collectively, this literature demonstrates that median nerve variation is clinically relevant but remains primarily descriptive, with limited standardized morphometric comparison across demographic groups.

Despite extensive descriptive reports on anatomical variation in the median nerve, quantitative morphometric analyses that integrate demographic and population-based comparisons remain limited. Hispanic populations, including Puerto Rican cohorts, have been largely underrepresented in cadaveric anatomical studies evaluating median nerve morphology. The absence of population-specific morphometric reference data limits assessment of whether clinically relevant differences may exist. This gap is crucial because anatomical assumptions derived from limited population samples may not accurately reflect the diversity of patients encountered in surgical and diagnostic practice. Although disparities in surgical outcomes among Hispanic and African American patients are multifactorial and largely related to socioeconomic and healthcare access factors [25], population-sensitive anatomical data may still support more precise procedural planning and imaging-based interpretation. Accordingly, standardized morphometric studies in underrepresented donor groups are needed to expand clinically applicable anatomical reference data.

Therefore, this study presents a multi-institutional cadaveric morphometric analysis of the median nerve to systematically characterize its formation, course, and distal branching patterns across defined upper-limb landmarks. Using standardized linear measurements, this study aims to (i) quantify segmental morphometric variation, (ii) evaluate differences associated with biological sex, and (iii) examine these patterns within a Puerto Rican cadaveric cohort that has not been previously characterized in the anatomical literature. By providing quantitative data from donor record population groups, this study expands current understanding of median nerve variability and contributes clinically relevant anatomical benchmarks for surgical planning, peripheral nerve repair, and imaging-based interpretation.

## 2. Methods

### 2.1. Cadaveric Procedure Approach

#### Cadaveric Specimens and Ethical Compliance

The Sam Houston State University College of Osteopathic Medicine Review Board reviewed and classified the study as exempt (IRB-2024-51). Eighty-two anatomical donors contributing paired bilateral upper limbs were examined through the Willed Body Programs at the University of Texas Health Science Center at Houston McGovern Medical School (*n* = 41 donors), Sam Houston State University College of Osteopathic Medicine (*n* = 22 donors), and the Universidad Central del Caribe School of Medicine (Puerto Rico) (*n* = 19 donors). Because each donor contributed both left and right upper limbs, the final dataset comprised 164 upper limbs (82 left and 82 right). No unilateral specimens were included. Donor age was not available in the de-identified institutional anatomical donation records and was therefore excluded from the statistical analyses (Table 1).

Donor demographic information, including biological sex and donor record population classification, was obtained from institutional anatomical donation records provided by the respective Willed Body Programs. For the purposes of this study, Puerto Rican, African American, and Caucasian were used as donor record population descriptors rather than genetically confirmed ancestry groups. Specimens obtained from the Universidad Central del Caribe School of Medicine represented donors from Puerto Rico. Consistent with U.S. Census Bureau/Office of Management and Budget classification, Puerto Rican origin is included within the Hispanic/Latino origin category, regardless of race [26]. However, Hispanic/Latino identity represents a heterogeneous demographic construct that may include diverse racial identities, ancestry backgrounds, and sociocultural histories [27]. Therefore, the Puerto Rican cohort was described as Hispanic for demographic context; however, this classification should not be interpreted as a genetic ancestry assignment.

All cadaveric specimens were preserved using standard formalin-based embalming protocols at their respective institutions prior to dissection. While minor variations in embalming composition and technique may exist between institutions, all specimens underwent conventional preservation methods commonly used in anatomical research. Because morphometric measurements were obtained under consistent dissection conditions and compared across similarly preserved specimens, any effects of tissue dehydration or fixation-related shrinkage are expected to be uniformly distributed across groups and therefore unlikely to systematically influence comparative analyses.

### 2.2. Dissection Protocol

Dissections were performed from the anterior compartment of the upper limb to ensure direct visualization of the median nerve from its origin in the brachial plexus (C5-T1) to its terminal branches in the hand. The skin, subcutaneous tissue, and superficial fascia were carefully reflected to expose the neurovascular bundle. The course of the median nerve was traced from its formation in the axilla, through the arm and forearm compartments, and into the carpal tunnel.

**Figure 2:**
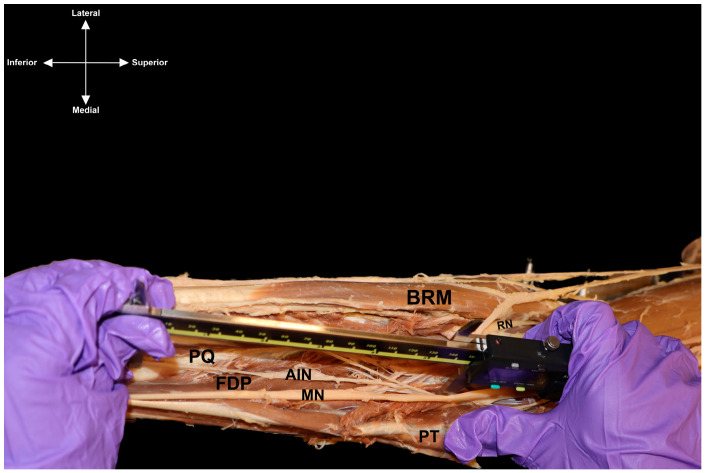
Morphometric measurement of the median nerve (MN) and its branches in the anterior compartment of the forearm. Representative cadaveric dissection illustrating the morphometric assessment of the MN using digital calipers to obtain linear measurements between defined anatomical landmarks. The MN is shown coursing through the anterior compartment of the forearm, with the anterior interosseous nerve (AIN) branching distally from the main nerve. The radial nerve (RN) is also identified laterally as part of the posterior compartment innervation pathway. Adjacent muscular landmarks include the brachioradialis muscle (BRM), flexor digitorum profundus (FDP), pronator quadratus (PQ), and pronator teres (PT).

Morphometric measurements were obtained using digital calipers (precision 0.01 mm) between predefined anatomical landmarks to ensure reproducibility. All measurements were performed with the limb positioned in anatomical orientation and with the median nerve fully exposed along its course (Figure 2). Measurements represent linear distances between predefined anatomical landmarks and do not reflect the true curvilinear length of the median nerve. The following landmarks were used for measurement:

- Lateral Cord Contribution (LC): distance from the point where the lateral cord emerges from the brachial plexus to its fusion with the medial cord forming the median nerve.
- Medial Cord Contribution (MC): distance from the origin of the medial cord to its point of fusion with the lateral cord at the formation of the median nerve.
- Medial Epicondyle of the Humerus (MEH): linear distance measured from the medial epicondyle to the midpoint of the median nerve as it traverses the cubital fossa.
- Lateral Epicondyle of the Humerus (LEH): linear distance from the lateral epicondyle to the midpoint of the median nerve at the level of the distal arm.
- Anterior Interosseous Nerve Origin (AIN): distance from the origin of the anterior interosseous nerve branching point to the proximal border of the pronator teres muscle.

All measurements were recorded in millimeters. To improve measurement reliability, each morphometric parameter was measured independently by two investigators, and the mean value was recorded for statistical analysis. In cases where measurements differed by more than 1 mm, a third verification measurement was performed.

### 2.3. Statistical Analysis

Quantitative measurements obtained from cadaveric dissections were analyzed to assess morphometric differences associated with biological sex and donor record population classification. Descriptive statistics were expressed as mean ± standard deviation (SD). Because each donor contributed paired bilateral upper limbs, mixed-effects modeling was used to account for repeated left- and right-side measurements within donors and potential imbalance in sample sizes among donor record population groups. Fixed effects included biological sex, donor record population classification, anatomical location, and side, where applicable. Anatomical location and side were treated as repeated factors within donors to account for the paired bilateral structure of the dataset. This modeling approach was selected to improve robustness of statistical inference in the presence of repeated measurements and unequal group sizes.

Site-specific morphometric comparisons were evaluated using paired or mixed-effects approaches according to the structure of each comparison. To control for potential inflation of Type I error associated with multiple comparisons, post hoc adjustments were applied using Tukey’s Honestly Significant Difference (HSD) correction. Because absolute morphometric measurements may be influenced by overall body size, proportional comparisons between selected anatomical landmarks were explored by calculating the ratio defined as the distance from the median nerve to the lateral epicondyle of the humerus (LEH) relative to the medial epicondyle of the humerus (MEH). These LEH/MEH ratios were analyzed separately for left and right limbs using unpaired two-tailed Welch-corrected *t*-tests to determine whether observed sex-related differences reflected relative anatomical positioning of the median nerve rather than global limb size variation.

Assumptions of normality and homogeneity of variance were evaluated prior to statistical testing. All statistical tests were two-tailed, and the level of significance was defined a priori at *p* < 0.05. Analyses were performed using GraphPad Prism (version 10.4; GraphPad Software, San Diego, CA, USA).

## 3. Results

### 3.1. Cadaveric Analysis

#### 3.1.1. Sex-Based Morphometric Differences

##### Proximal Formation

**Figure 3:**
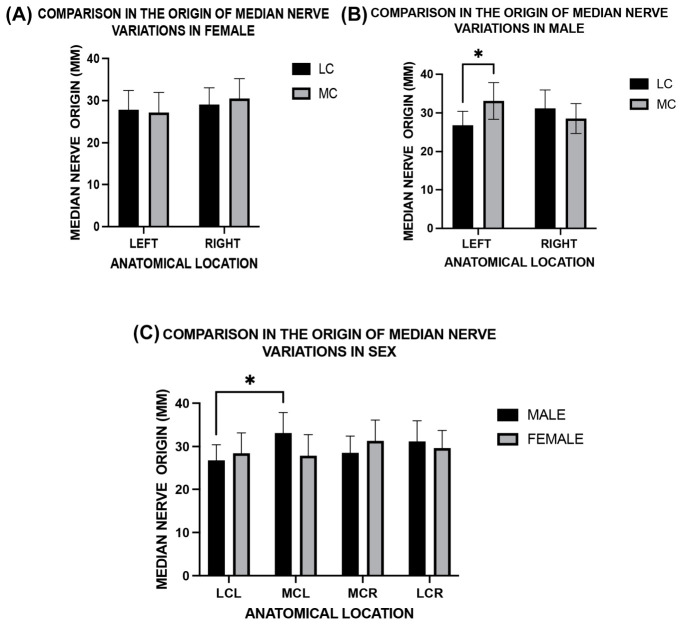
Morphometric comparison of median nerve (MN) formation by biological sex and cord contribution. (**A**) Mean length (± SD) of the lateral cord (LC) and medial cord (MC) contributions forming the MN in female upper limbs, showing no statistically significant differences between cords or sides (*p* > 0.05). (**B**) Corresponding analysis in male specimens demonstrated a significant difference on the left side, where the MC contribution was longer than the LC contribution (* *p* = 0.0472), while no significant difference was observed on the right side (*p* = 0.396). (**C**) Combined comparison of cord contribution lengths between males and females across left and right anatomical locations (* *p* = 0.0239). LCL = left lateral cord; MCL = left medial cord; MCR = right medial cord; LCR = right lateral cord. Asterisk indicates statistical significance.

At the brachial plexus origin of the median nerve, morphometric differences were identified between sexes and between contributing cords (Figure 3). Female specimens did not exhibit significant inter-cord or side-dependent differences, nor significant differences between left and right cord contribution measurements (*p* = 0.60) (Figure 3A). The mean length of the LC and MC contributions forming the median nerve in female specimens was 28.86 ± 11.3 mm. In contrast, male specimens demonstrated a statistically significant difference between cord contributions on the left side (Figure 3B). The MC contribution measured a longer cord segment than the LC contribution to the formation of the median nerve (33.11 mm vs. 26.80 mm; t(30) = 2.38; adjusted *p* = 0.0472). No statistically significant difference between cords was observed on the right side (*p* = 0.396).

When LC and MC contribution lengths were compared between sexes (Figure 3C), males exhibited a greater mean value than females (29.91 ± 11.49 mm vs. 28.85 ± 10.12 mm). However, this difference did not reach statistical significance (F(1, 30) = 0.11; *p* = 0.74). The mixed-effects model demonstrated significant within-subject matching across anatomical measurements (χ^2^ = 48.77, df = 3, *p* < 0. 0.0239), indicating consistency in the measurements despite moderate within-group variability.

##### Forearm Distribution

**Figure 4:**
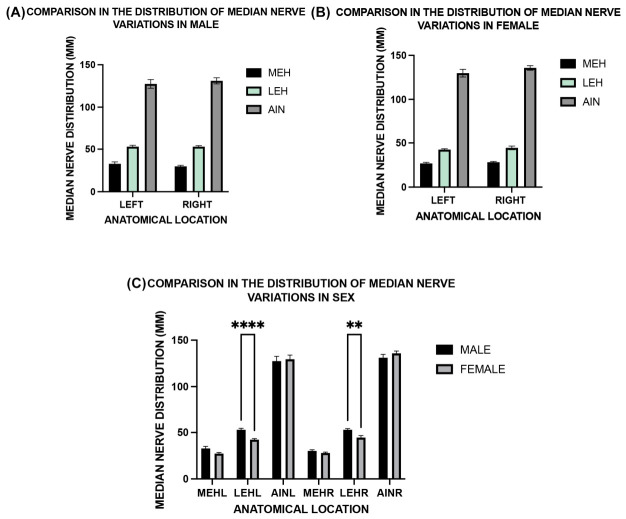
Landmark-based morphometric distribution of the median nerve (MN) across forearm anatomical landmarks by biological sex. (**A**) Mean landmark-based morphometric distances (± SD) of MN measured in male upper limbs at three anatomical landmarks: medial epicondyle of the humerus (MEH), lateral epicondyle of the humerus (LEH), and the origin of the anterior interosseous nerve (AIN) on both left and right sides. (**B**) Corresponding measurements in female specimens demonstrating a comparable distribution across anatomical locations. (**C**) Combined comparison of MN landmark-based morphometric distances between males and females across bilateral anatomical landmarks. MEHL = medial epicondyle of the humerus, left side; LEHL = lateral epicondyle of the humerus, left side; AINL = anterior interosseous nerve, left side; MEHR = medial epicondyle of the humerus, right side; LEHR = lateral epicondyle of the humerus, right side; AINR = anterior interosseous nerve, right side. Asterisks indicate statistically significant differences between sexes (** *p* < 0.01; **** *p* < 0.0001).

Morphometric analysis of the forearm segment of the median nerve demonstrated significant variation across anatomical locations in both male and female cohorts (Figure 4). In the male cohort, mixed-effects modeling revealed a significant main effect of anatomical location on measured morphometric distance (F(1.21, 72.85) = 663.8; *p* < 0.0001) (Figure 4A). This pattern reflected increasing linear distances between the defined anatomical landmarks (MEH, LEH, and AIN). No statistically significant effect of laterality or interaction between side and anatomical location was detected.

Similarly, analysis of female specimens demonstrated a significant effect of anatomical location (F(1.29, 38.75) = 907.3; *p* < 0.0001) (Figure 4B). As in males, neither laterality nor interaction terms were statistically significant, indicating comparable left-right distributions of measured morphometric distances. Post hoc comparisons within the male cohort identified one statistically significant contrast between proximal landmarks: the left LC and MC contributions differed by −6.31 mm (95% CI −11.72 to −0.89; *p* = 0.0239; adjusted *p* = 0.0472). No significant within-sex contrasts were detected in the female cohort.

When both sexes were analyzed together in a combined mixed-effects model (Figure 4C), a significant main effect of anatomical location was again observed (F(2.67, 80.13) = 635.4; *p* < 0.0001). The overall effect of biological sex did not reach statistical significance (F(1, 30) = 4.16; *p* = 0.0502). Mean predicted measured distance was 71.25 mm in males and 67.88 mm in females (Δ = 3.37 mm; 95% CI −0.003 to 6.742). Pairwise comparisons indicated that measured morphometric distances at the lateral epicondyle of the humerus on both the left (LEHL) and right (LEHR) sides were greater in males than in females (*p* < 0.0001 for both comparisons).

To determine whether sex-related differences in absolute morphometric measurements were influenced by overall limb size, normalized LEH/MEH ratios were calculated for each limb. Comparison of these ratios between male and female specimens revealed no statistically significant difference on either side of the body (left: males 1.504 ± 0.3329 vs. females 1.505 ± 0.3313, *p* = 0.9927; right: males 1.913 ± 0.5171 vs. females 1.856 ± 0.4572, *p* = 0.7213; Welch-corrected *t*-test) (Table 2).

##### Population Group Variation

**Figure 5:**
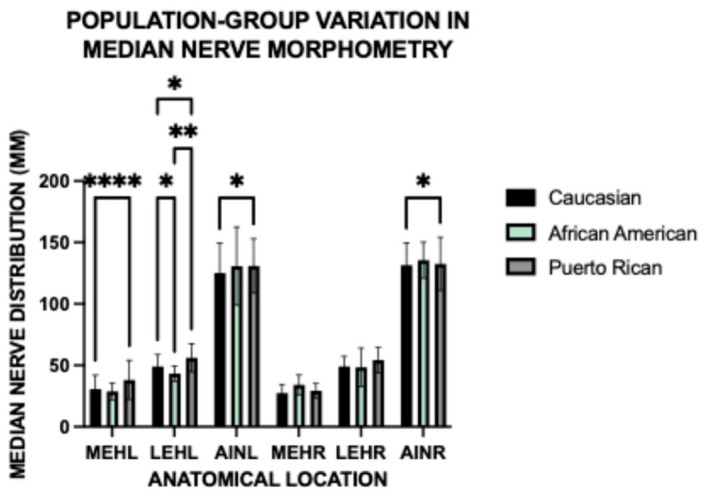
Population group variation in landmark-based median nerve (MN) morphometry across upper-limb regions. Comparison among donor record Puerto Rican, Caucasian, and African American groups revealed significant population group differences in measured morphometric distances (* *p* < 0.05; ** *p* < 0.01; **** *p* < 0.0001), with the Puerto Rican cohort exhibiting the highest mean values across anatomical landmarks.

Population group comparisons were performed by pooling measurements across biological sex to evaluate overall morphometric differences among donor record Puerto Rican, Caucasian, and African American cohorts; sex-specific effects were analyzed separately in prior models. Mixed-effects modeling revealed a significant main effect of population group (F(2.74, 128.8) = 468.2; *p* < 0.0001). A smaller but statistically significant effect of anatomical location was also detected (F (0.90, 42.27) = 4.768; *p* = 0.0382). The interaction between population group and anatomical location was not statistically significant (*p* = 0.1661). Descriptive analysis demonstrated that the Puerto Rican cohort exhibited greater mean morphometric distances between defined anatomical landmarks compared with the Caucasian and African American cohorts (Figure 5). These differences were observed across both proximal and distal anatomical sites. Variance analysis demonstrated lower within-group dispersion in the Caucasian sample (SD = 1.923 mm) relative to the Puerto Rican and African American cohorts.

## 4. Discussion

This multi-institutional cadaveric investigation provides quantitative evidence that median nerve morphometry varies across defined anatomical landmarks and differs according to biological sex and donor record population classification. Three principal findings emerged. First, sex-related morphometric differences were localized primarily to the proximal upper limb, particularly at the level of median nerve formation and proximal forearm landmarks. Second, measured distances increase across proximal-to-distal anatomical landmarks; however, these values represent linear distances between predefined reference points rather than the true curvilinear length. Therefore, this pattern should be interpreted as reflecting the spatial distribution of selected anatomical landmarks rather than intrinsic elongation of the median nerve. Third, population group comparisons identified greater measured morphometric distances in donors classified as Puerto Rican compared with donors classified as Caucasian or African American. Together, these findings provide population-sensitive morphometric data that may improve anatomical interpretation during surgical planning and diagnostic imaging of the upper limb.

Previous anatomical investigations have reported median nerve variability at multiple levels of the upper limb. Proximally, accessory roots and atypical fusion patterns between the lateral and medial cords have been described in South Asian and Russian populations, with reported supernumerary root formation ranging from approximately 15% to 33% in selected cadaveric series [3,5,6,19,20]. These findings are consistent with the present observation that sex-related differences were most apparent near the proximal formation of the nerve. Such variability is clinically relevant because alterations in cord contributions may alter the spatial relationship between the median nerve and adjacent axillary vessels, potentially increasing the risk of iatrogenic injury during axillary surgery or regional anesthesia.

In the forearm, reported variants include penetration of the flexor digitorum superficialis, high bifurcation, and atypical fascicular distributions, each of which may alter the nerve’s relationship to muscular and fascial structures [23,24,28,29,30,31,32,33]. Distally, recurrent thenar motor branch variation, bifid median nerve, persistent median artery, and trifurcation patterns remain clinically important because they may increase the risk of incomplete decompression or iatrogenic injury during carpal tunnel release [13,15,18,21,22,34,35,36,37,38,39,40,41,42,43,44,45]. The present study complements this literature by providing quantitative morphometric data across defined landmarks, rather than relying solely on qualitative descriptions of branching patterns.

The present study also identified localized sexual dimorphism in median nerve morphology. Male specimens demonstrated significantly longer medial cord contributions to the formation of the median nerve on the left side, whereas female specimens exhibited relatively symmetrical cord lengths. Although overall arm-level nerve length did not differ significantly between sexes, statistically significant differences were observed at proximal landmarks near the lateral epicondyle. These findings are consistent with prior reports describing sex-related differences in upper limb musculoskeletal architecture and neurovascular organization, which may influence peripheral nerve trajectory and mechanical loading patterns.

Because absolute morphometric measurements may be influenced by overall body size, exploratory normalization was performed by calculating the LEH/MEH ratio to evaluate the relative positioning of the median nerve between epicondylar landmarks. No significant differences in this ratio were observed between male and female donors on either side. These findings suggest that absolute differences observed in raw morphometric measurements may reflect overall limb-size scaling rather than a proportional shift in the anatomical course of the median nerve. This supports the interpretation that median nerve positioning remains relatively consistent across sexes when normalized to adjacent skeletal landmarks.

Population group comparisons further demonstrated that donors classified in institutional records as Puerto Rican exhibited greater mean morphometric distances across multiple anatomical landmarks compared with donors classified as Caucasian and African American. These categories should be interpreted as donor record population descriptors rather than genetically confirmed ancestry groups. While previous anatomical studies have primarily focused on qualitative branching variants, quantitative differences in peripheral nerve morphometry across demographically defined cohorts have been less frequently reported. The present findings, therefore, contribute novel morphometric data suggesting that population-sensitive anatomical reference ranges may be valuable when interpreting peripheral nerve measurements. However, because donor record classifications may not fully capture genetic ancestry, admixture, or environmental contributors to morphology, these findings should be interpreted cautiously and validated in larger, prospectively characterized cohorts. Establishing such reference ranges may ultimately improve interpretation of morphometric measurements obtained through ultrasound and MR neurography.

Developmentally, these anatomical differences may reflect variability in the processes governing brachial plexus formation and peripheral nerve elongation. During early limb development, motor axons extend distally from the cervical spinal cord through the mesenchymal limb bud under the influence of signaling pathways including HOX gene regulation, fibroblast growth factors, and sonic hedgehog gradients [1]. Variations in the timing of axonal convergence or cord fusion may alter the position at which lateral and medial cord fibers merge to form the median nerve. Differential limb growth during fetal development may also contribute to elongation of nerve segments relative to surrounding musculoskeletal structures, potentially explaining the morphometric asymmetries observed in the present study.

From a diagnostic perspective, recognition of these morphometric patterns has important implications for imaging-based evaluation of the median nerve. High-resolution ultrasound is widely used to assess peripheral nerve morphology, particularly in the diagnosis of carpal tunnel syndrome and proximal compression neuropathies [11,46,47,48,49,50]. Variation in measured nerve dimensions and branching configuration may influence sonographic measurements such as cross-sectional area and fascicular organization. Awareness of such anatomical variability may therefore assist clinicians in distinguishing pathological nerve enlargement from normal anatomical variation.

Clinically, these findings support population- and sex-aware planning for surgical procedures involving the upper limb. Procedures performed near the distal humerus, pronator teres, and proximal forearm require careful consideration of median nerve trajectory and potential branching anomalies. Similarly, accessory roots or asymmetrical cord contributions may complicate axillary or infraclavicular nerve block procedures, reinforcing the importance of ultrasound-guided identification of individual cord structures during regional anesthesia [2,3,5,24]. Recognition of distal branching variants is particularly important during carpal tunnel release, where injury to the recurrent motor branch may result in persistent thenar dysfunction or require repair [13,15,34,36,37,38,39,40].

Despite the strengths of this multi-institutional cadaveric dataset, several limitations should be acknowledged. The unequal distribution of specimens across biological sex and donor record population groups, including a lower representation of female donors, may influence statistical power and limit the ability to fully distinguish biological variation from sampling effects. Although mixed-effects modeling was used to account for unbalanced sample sizes and repeated bilateral measurements, the observed sex- and population-related differences should be interpreted as cohort-specific morphometric patterns rather than definitive population-level characteristics. In addition, the population categories used in this study were based on institutional donor records and should not be interpreted as genetically confirmed ancestry groups. Terms such as Puerto Rican, African American, and Caucasian reflect demographic classifications available through donor documentation and may encompass heterogeneity in ancestry, admixture, socioeconomic background, and environmental exposures. Donor age, limb dominance, occupational history, and comorbid conditions were not available and may influence peripheral nerve morphology. Finally, morphometric assessment was restricted to gross anatomical measurements and did not include histological evaluation of fascicular organization or axonal density. Future investigations integrating larger balanced cohorts, imaging-based morphometry, and histological analysis may further clarify the structural organization of the median nerve across diverse populations.

## 5. Conclusions

This multi-institutional cadaveric study provides quantitative morphometric evidence that median nerve anatomy demonstrates measurable variation across defined upper-limb landmarks, with observed differences associated with biological sex and donor record population classification. The principal contribution of this work is the generation of standardized, landmark-based morphometric data from a demographically diverse cadaveric sample that includes a Puerto Rican cohort, a group previously underrepresented in median nerve anatomical research.

Importantly, the measurements reported in this study represent linear distances between predefined anatomical landmarks rather than true curvilinear nerve length. This distinction strengthens methodological interpretation and supports reproducibility in future morphometric and imaging-based studies. Clinically, these data provide reference values that may inform surgical planning, peripheral nerve repair, regional anesthesia, and diagnostic assessment of median nerve pathology. Future investigations using larger and more balanced cohorts, high-resolution imaging, and histological analysis are warranted to validate these findings and further define population-sensitive anatomical reference standards.

## Figures and Tables

**Table 1:** Donor and upper-limb specimen characteristics.

Donor Record Population Classification	Male Donors, *n*	Female Donors, *n*	Total Donors, *n*	Total Upper limbs, *n*	Side Distribution
Puerto Rican	13	6	19	38	19 left/19 right; 0 unilateral
Caucasian	37	12	49	98	49 left/49 right; 0 unilateral
African American	10	4	14	28	14 left/14 right; 0 unilateral
Total	60	22	82	164	82 left/82 right; 0 unilateral

**Table 2:** Normalized LEH/MEH ratios by sex.

Sex	Left Ratio (Mean ± SD)	Right Ratio (Mean ± SD)
Male	1.504 ± 0.3329	1.913 ± 0.5171
Female	1.505 ± 0.3313	1.856 ± 0.4572
*p* value	0.9927	0.7213

LEH/MEH ratios were calculated separately for left and right upper limbs. Values are presented as mean ± standard deviation (SD). Comparisons were performed using unpaired two-tailed Welch-corrected *t*-tests (left: *p* = 0.9927; right: *p* = 0.7213).

## Data Availability

The data supporting the findings of this study are available from the corresponding author upon reasonable request. The data are not publicly available because they were derived from de-identified cadaveric donor records and anatomical specimens obtained through institutional Willed Body Programs, which are subject to ethical and institutional data-use restrictions.
